# Active Substances from the Micro-Immunotherapy Medicine 2LMIREG Display Antioxidative Properties In Vitro in Two Colorectal Cancer Cell Lines

**DOI:** 10.3390/life15050743

**Published:** 2025-05-06

**Authors:** Laura Garcia-Sureda, Camille Jacques, Daniel G. Pons, Jorge Sastre-Serra, Jordi Oliver, Ilaria Floris

**Affiliations:** 1 Preclinical Research Department, Labo’life España, 07330 Consell, Spain; 2 Preclinical Research Department, Labo’life France, Pescalis-Les Magnys, 79320 Moncoutant-sur-Sevre, France; ilaria.floris@labolife.com; 3 Grupo Multidisciplinar de Oncología Traslacional, Institut Universitari d’Investigació en Ciències de la Salut (IUNICS), Universitat de les Illes Balears, 07122 Palma de Mallorca, Spain; d.pons@uib.es (D.G.P.); jorge.sastre@uib.es (J.S.-S.); jordi.oliver@uib.es (J.O.); 4 Instituto de Investigación Sanitaria Illes Balears (IdISBa), Hospital Universitario Son Espases, Edificio S, 07120 Palma de Mallorca, Spain; 5 Ciber Fisiopatología Obesidad y Nutrición (CB06/03), Instituto Salud Carlos III, 28029 Madrid, Spain

**Keywords:** micro-immunotherapy, mitochondria, HT-29 cell line, SW620 cell line, in vitro, reactive oxygen species, colorectal cancer

## Abstract

Mitochondria play a crucial role in oxidative stress control and reactive oxygen species (ROS) generation, impacting many cellular processes. Dysregulated mitochondria are linked to diseases such as colorectal cancer (CRC), known for its aggressiveness. Since ROS plays a role in tumor growth and metastasis, there is considerable interest in developing therapies that target these reactives. This study investigates the effects of some active substances from the micro-immunotherapy (MI) medicine 2LMIREG^®^ on mitochondrial metabolism parameters in two CRC-derived cell lines. HT-29 and the metastasis-derived SW620 cell lines, which heavily rely on ROS for proliferation, were used to evaluate the effects of the tested active substances. Cellular viability and various mitochondrial metabolism parameters were measured: ROS production, mitochondrial mass index, and mitochondrial DNA levels. In both cell lines, the tested MI formulation reduced cellular viability as well as ROS production compared to the vehicle used as a control. The treatment also appeared to increase the mitochondrial mass index without affecting mitochondrial DNA levels in the two CRC models. Altogether, these preliminary results report for the first time the mitochondria-related effects of some actives from 2LMIREG^®^ in two CRC cell models and open perspectives for further in-depth metabolism-based studies.

## 1. Introduction

Colorectal cancer (CRC) is a serious public health problem worldwide, classified as the third most common cancer and the second cancer causing death [1]. Its impact on morbidity and mortality underscores the urgent need for increased awareness, early detection, and effective intervention strategies.

Reactive oxygen species (ROS) are normal mitochondria metabolism byproducts, and this term refers to oxygen free radicals such as superoxide anion radical (O^2·−^), hydroxyl radical (·OH), etc. Oxidative stress and mitochondrial dysfunction play crucial roles in cancer development and progression, particularly in CRC, in which ROS levels were reported to be more elevated than in normal cells [2]. Indeed, any imbalance between ROS production and the cellular antioxidant defense mechanisms can promote tumor initiation, progression, and resistance [3,4,5]. Mitochondria, as the main producers of cellular energy, are intricately involved in maintaining redox homeostasis and regulating apoptotic pathways. Dysfunction in mitochondrial respiration and metabolism can increase ROS production and genomic instability, and alter signaling pathways, thus contributing to CRC development [6]. Thus, in any healthy cell, the maintenance of redox homeostasis is a critical factor, and keeping proper function of the antioxidant systems is of paramount importance [7].

Accumulating evidence has attributed to ROS a “double-edged sword” role in cancer cells. When their levels are relatively low, ROS act as stimuli able to activate cancer cell proliferation, migration, invasion, angiogenesis, and drug resistance [8]. On the other hand, if ROS levels are too elevated, they can also promote cancer cell senescence and cell death [9]. For instance, in CRC, Wu et al. recently showed that the combination of isoalantolactone and doxorubicin displayed cancer cell cytotoxicity through increased ROS and deoxyribonucleic acid (DNA) damage accumulation [10]. However, concerning the bidirectional nature of ROS, there is also an opposite strategy aimed at downregulating ROS levels in cancer cells to induce cell death [11,12]. For this concern, it has been observed that antioxidants could be useful for both cancer prevention and treatment, including CRC [13,14]. The duality of ROS in the context of CRC can explain why two opposite strategies can be employed: either a pro-oxidant or an anti-oxidant therapy [15]. In the meantime, the complex interplay between ROS levels and CRC physiology makes it difficult to achieve efficient and safe therapeutic solutions, with minimal side effects. Further research studies are ultimately needed in this field.

Micro-immunotherapy (MI) medicinal products from Labo’life, also referenced as MI medicines (MIMs) in the literature, show promising preclinical results in the treatment of both acute and chronic diseases, for instance, in inflammatory illnesses such as influenza A infection [16], arthritis [17,18,19], periodontitis [20,21,22], and allergies [23,24], in which in vitro and in vivo anti-inflammatory effects were observed, both locally and systemically [25]. In addition, the biological effects and the potential of these medicinal products as adjuvant medicines in the context of cancers have previously been investigated both in vitro, in 2D and 3D models of CRC, and in vivo [26]. Labo’life MIMs employ low doses (LDs) and ultra-low doses (ULDs) of immune factors (mostly cytokines) in association with nucleic acids (plant-derived total DNA and ribonucleic acid (RNA)) or specific nucleic acids (SNA^®^), as well as other bioactive molecules, to regulate immune responses and sustain the immune system in several pathological contexts. These active ingredients at LDs/ULDs, mainly expressed in centesimal Hahnemannian dilutions (CHs), are reached through serial dilutions, also called serial kinetic process, and are further impregnated on sugar pillules, also called globules, for oromucosal administration [16,20]. One more particularity of most of the MIM resides is the fact that they are sequential medicines made of different capsules containing pillules having a specific composition.

In the present research, we focused on a particular combination of active substances employed in the sequential MIM 2LMIREG^®^, specifically in the 10th capsule. This capsule, hereafter referred to as MIM-10, has previously been shown able to modulate immune functions and reduce oxidative stress by decreasing ROS production in vitro in a model of human-derived peripheral blood mononuclear cells (PBMCs) [27]. To pursue the investigation of MIM-10, we assessed its effect on ROS production and several mitochondria-related features in two cellular models of CRC, the HT-29 cells and the metastatic SW620 cells, which are well known for their aggressive properties [28]. Indeed, based on the mitochondria-related effects displayed by MIM-10 in immune cells [27], we wanted to investigate its effects in CRC cell lines known for their ROS-dependent invasive phenotypes, as CRC cell lines can use ROS signaling to sustain cell migration and invasion [29,30]. While an in vitro model does not replicate the complex system of the tumor environment, the cell lines used in this study could also be used to study the mitochondria and redox balance of CRC [29,30].

This preliminary study aimed to analyze the effect of the aforementioned MIM-10 capsule in HT-29 and SW620 cell lines by assessing cell viability, ROS production, and other mitochondrial functional parameters [mitochondrial mass and mitochondrial DNA (mtDNA) levels, metabolic enzyme activities like cytochrome-c oxidase (COX), and citrate synthase (CS)] as aerobic mitochondrial metabolism indicators, as well as lactate dehydrogenase (LDH) as an anaerobic metabolism indicator.

## 2. Materials and Methods

### 2.1. Chemicals and Reagents

Dulbecco’s modified Eagle’s medium (DMEM) with high glucose was from Gibco (Paisley, UK). Fetal bovine serum (FBS) and penicillin–streptomycin were from Biological Industries (Kibbutz Beit Haemek, Israel). Dimethyl sulfoxide (DMSO), Tween-20, TRI Reagent^®,^ and Hoechst 33342 were from Sigma–Aldrich (St. Louis, MO, USA). Protein determination was performed using the bicinchoninic acid (BCA) method, thanks to the BCA^TM^ Protein Assay Kit from Pierce (Bonn, Germany). Human Western blot antibodies SIRT1 (sc-15404) and Tubulin (sc-5286) were from Santa Cruz Biotechnology (Texas, CA, USA), and the total OXPHOS human WB antibody cocktail (#MS601) was from MitoSciences^®^ (Eugene, OR, USA). Immun-Star# Western C# Chemiluminescent Kit and Trans-Blot Turbo Mini Nitrocellulose Transfer Packs were from Bio-Rad Laboratories (Hercules, CA, USA). Finally, Amplex^VR^ Red Reagent and MitoTracker^VR^ Green (MTG) were from Invitrogen—Molecular Probes—Thermo Fisher Scientific (Waltham, MA, USA).

### 2.2. Investigational Product

The tested formulation corresponds to the combination of active substances at ULDs impregnated on sucrose-lactose pillules, or globules, and encapsulated in capsule 10 (the 10th capsule of the blister) of the sequential MI medicine 2LMIREG^®^ (thus also called MIM-10) manufactured by Labo’life España, as previously described [23,31]. Its composition is as follows: recombinant human (rh)-transforming growth factor beta (TGF-β) at 15 CH, rh-interleukin (IL)-1β at 10 CH, rh-IL-2 at 10 CH, rh-IL-5 at 10 CH, rh-IL-6 at 10 CH, rh-tumor necrosis factor-alpha (TNF-α) at 10 CH, prostaglandin E2 (PGE2) at 10 CH; DNA at 10 CH, RNA at 10 CH, SNA^®^-HLA I at 10 CH, and SNA^®^-HLA II at 10 CH, developed to target human leukocyte antigens (HLAs) I and II, respectively, and finally SNA^®^-MIREG at 16 CH, intended to target human IL-2.

The sequential MI medicine 2LMIREG^®^ (composed of ten different capsules: MIM-1, MIM-2, MIM-3, MIM-4, etc.) has been tested according to the order of administration to assess its effect on cell viability and ROS production (see Section 2.5 and Section 2.6). Since no effects were found on these readouts (see the results in Supplementary Figure S1), the study focused only on MIM-10.

The vehicle (Veh.), used as a control in this study was manufactured as previously described [20,31]. Briefly, it consists of lactose–sucrose pillules lacking active substances and provides a suitable experimental control for the preclinical research studies.

To treat the cells, the media were prepared by dissolving the globules contained in 1 capsule (380 mg) of Veh., or MIM-10 in 100 mL of media. This corresponds to a final sucrose–lactose concentration of 11 mM.

### 2.3. Human Cell Lines

The non-metastatic human CRC cell line HT29 (derived from a primary colorectal adenocarcinoma) and the metastatic SW620 (derived from the lymph node of metastatic colorectal tumor) were purchased from American Type Culture Collection (ATCC, Manassas, VA, USA) and maintained in DMEM supplemented with 10% (vol/vol) of heat-inactivated FBS and 1% (vol/vol) of antibiotics cocktail (penicillin and streptomycin) at 37 °C with 5% CO_2_. Cells were seeded and treated as described in the next section. The experiment was run in six sample replicates (*n* = 6) for each group.

### 2.4. Cell Treatment with the Tested Item MIM-10

**Figure 1:**
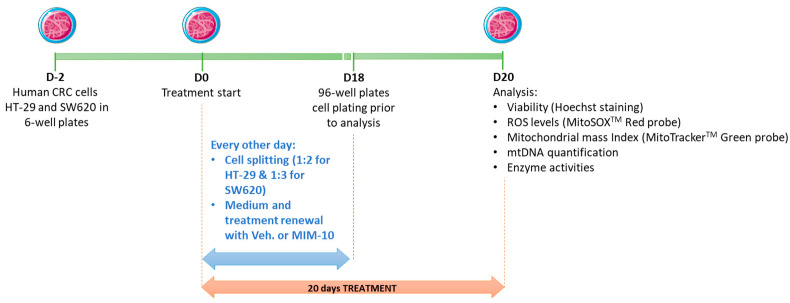
Representative scheme of the experimental protocol. The non-metastatic HT-29 cells and the metastatic lymph node-derived cells of the SW620 cell line, at confluence (D0), were treated for 20 days with either the Veh. or the MIM-10. Briefly, the cells were splitted every other day (1:2 for HT-29 cells and 1:3 for SW620 cells) using freshly prepared media. Cell viability, ROS levels, mitochondrial mass, and enzyme activities were further assessed at day 20. CRC: colorectal cancer; MIM: micro-immunotherapy medicine; mtDNA: mitochondrial DNA; ROS: reactive oxygen species; Veh.: vehicle.

A blind in vitro experiment was performed with either the Veh. or the MIM-10 treatment. As depicted in the schema provided in Figure 1, HT-29 and SW620 cells were initially seeded in six-well plates at a density of 1.2 × 10^6^ cells/well and 6 × 10^5^ cells/well, respectively, and incubated for 48 h. On day 0 (D0), treatment started with either the Veh. or the MIM-10. To assess the effect of a long exposure lasting 20 days, maintaining optimal cell culture conditions, both cells were split and plated again in new six-well plates every other day. Considering the different proliferative capacities, the ratio differs among them. HT-29 cells were split 1:2, while 1:3 was the ratio used for SW620. On the last day of subculture and treatment (D18), cells were plated in 96 wells for two days more of treatment before the analysis.

### 2.5. Cell Viability Assay

Cell viability was determined by fluorometric assay staining the DNA with Hoechst 33342 (Sigma-Aldrich, St. Louis, MO, USA; 350 nm/455 nm, excitation and emission wavelengths, respectively), as described before [32]. The data are expressed as a percentage relative to the Veh. group, whose average of replicates was set at 100%.

### 2.6. ROS Production

Reactive oxygen species production levels were determined by a fluorometric assay using a MitoSOX^TM^ Red probe (Invitrogen, Carlsbad, CA, USA). The assay employed 430 nm/590 nm, excitation, and emission wavelengths, respectively, as described before [33]. The data are expressed as a percentage relative to the Veh. group, whose average was set at 100%.

### 2.7. Index of Mitochondrial Mass

Mitochondrial mass was determined by a fluorometric assay using MitoTracker^®^ Green probe [33]. The data are expressed as a percentage compared to the Veh. group, whose average was set at 100%.

### 2.8. mtDNA Quantification

Total DNA was isolated from cultured cells using TRI Reagent^®^ following the manufacturer’s protocol and then quantified using a BioSpec-nano spectrophotometer (Shimadzu Biotech, Kyoto, Japan) set at 260 nm. mtDNA was semi-quantified using quantitative reverse transcription-polymerase chain reaction (qRT-PCR) method as described before [33]. Quantitative RT-PCR was performed using SYBR Green technology on a Light-Cycler 480 System II rapid thermal cycler (Roche Diagnostics, Basel, Switzerland). The data is calculated as the relative expression with respect to the Veh. group, set as 1.

### 2.9. Enzymatic Activities

After 20 days of the treatment, cells were harvested by scraping them into 200 μL of STE buffer (250 mM sucrose, 3.59 mM Trizma-Base, 16.4 mM Tris-HCl pH 7.4, 2 mM EDTA, 40 mM KCl). Then, cells were disrupted by sonication at 40% amplitude for 10 s, three times (VibraCell 75185, Sonics & Material, Newtown, CT, USA) and centrifuged at 600× *g* for 10 min at 4 °C to remove cell debris. Protein content within the supernatants was determined with a bicinchoninic acid (BCA) protein assay kit (Pierce, Bonn, Germany), and the enzymatic assays were performed immediately after. Lactate dehydrogenase (LDH), citrate synthase (CS), and cytochrome-c oxidase (COX) enzymatic activities were determined using spectrophotometric methods as described before [33,34,35]. The data are expressed as mU/mg of protein.

### 2.10. Statistics

The graphs in the figures were performed using GraphPad Prism for macOS (version 10.3.1, updated 21 August 2024, GraphPad Software, La Jolla, CA, USA, www.graphpad.com). The authors have followed the recent recommendations of D.L. Vaux [36] that encourage performing descriptive statistics instead of making statistical inferences when the number of independent values is small. In the case of results derived from only one, two, or three (*n* = 1, *n* = 2, or *n* = 3) in vitro experiment(s), it is recommended to include a full dataset, plotting data points, letting the readers interpret the data for themselves, rather than drawing statistical inferences, showing *p* values, standard error of the mean (S.E.M.), or results that are not representative. Indeed, no statistical inference has been performed to analyze the results presented here, as one single experiment was run in six replicates. Thus, the graphs in the figures show all replicates, the mean, and the standard deviation (S.D.).

## 3. Results

### 3.1. MIM-10 Slightly Reduced the Viability and Diminished the Reactive Oxygen Species Production Within the HT-29 Cells and the Metastatic Colon Cancer SW620 Cells

The present study aimed to assess the effects of MIM-10 on the cellular viability of two CRC models, the HT-29 cell line, and the metastatic SW620 cell line, which is highly invasive. As described in the Materials and Methods section, the cancer cells were treated with either MIM-10 or the vehicle (Veh.) for 20 days before performing a complete endpoint analysis of several parameters related to cell viability and mitochondria metabolism (Figure 1).

**Figure 2:**
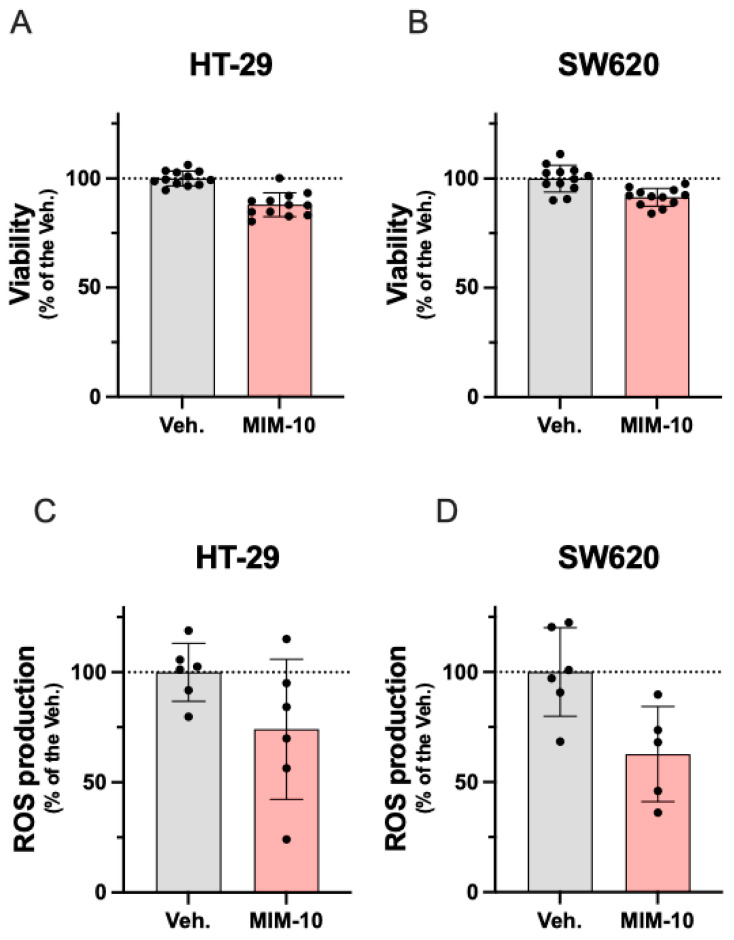
Effect of a 20-day MIM-10 treatment on cell viability (**A**,**B**) and mitochondrial ROS production (**C**,**D**) in a non-metastatic HT-29 CRC cell line and metastatic SW620 CRC cell line**.** Data are presented as percentages relative to the Veh. group, in which the average of replicates was set at 100%. Data represent means ± S.D. from *n* = 12 replicates (**A**,**D**) and *n* = 6 replicates (**B**,**D**). The dotted lines are drawn to highlight the effects of MIM-10 compared with the Veh. treatment.

As a first readout, the effect of MIM-10 on cell viability was measured at different time points (on day 4, day 12, day 16, and day 20) using Hoechst staining. As shown in Supplementary Figure S2, viability was not affected or just barely reduced compared to the untreated control at earlier time points. On day 20, as Figure 2A,B shows, MIM-10 reduced by about 10% the viability of both CRC cells compared with the Veh. control group. The effects of MIM-10 on reactive oxygen species (ROS) production were evaluated by MitoSOX^TM^ Red probe. As shown in Figure 2C,D, MIM-10 decreased ROS production after 20 days of treatment in both cell lines by about 40%, compared to the Veh.-treated cells.

Overall, this first body of data, while still preliminary, suggests the potential effect of MIM-10 in slightly impairing the viability of CRC cells, possibly linked with its inhibitory effect on ROS production.

### 3.2. MIM-10 Slightly Increased the Mitochondrial Mass Index Without Affecting the Mitochondrial DNA Level Within the HT-29 Cells and the Metastatic SW620 Cells

**Figure 3:**
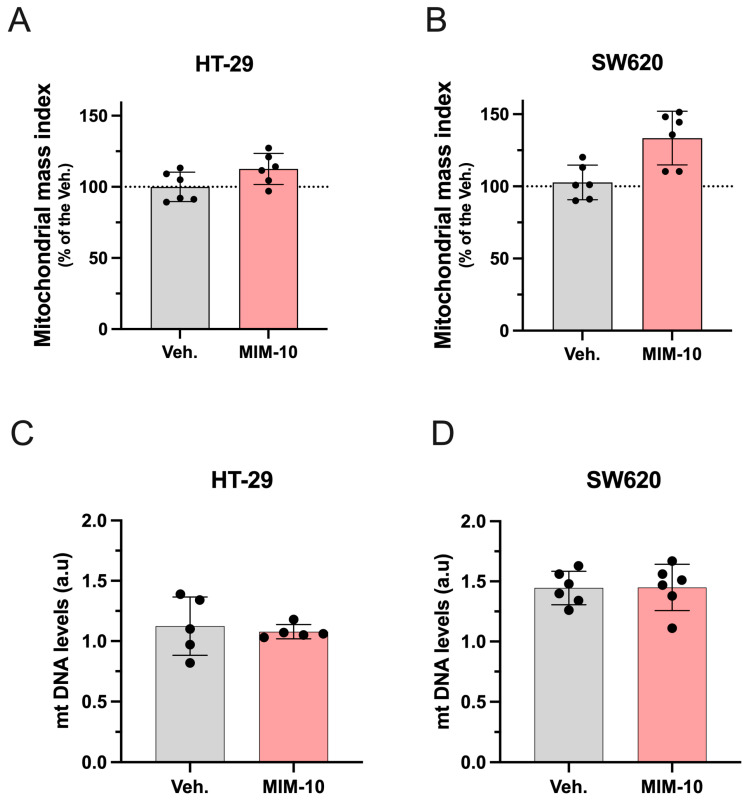
Effect of 20-day MIM-10 treatment on the mitochondrial mass index in HT-29 (**A**) and SW620 (**B**) colon cancer cell lines. Mitochondrial mass index is expressed as a percentage relative to the Veh. group, whose average is set at 100%. Data are presented as means ± S.D. from *n* = 6 replicates. The dotted lines are drawn to highlight the effects of MIM-10 in comparison to the Veh. treatment. The effect on relative mtDNA levels in HT-29 (**C**) and SW620 (**D**) is expressed in arbitrary units (a.u).

To further analyze the effect of MIM-10 on mitochondria-related functions and metabolism, the mitochondrial mass index was assessed by employing MitoTracker^VR^ Green (MTG) as a fluorescent probe specifically designed to target mitochondria. Figure 3A,B shows that MIM-10-treated HT29 and SW620 cells both displayed an increment of the mitochondria mass of about 20% in comparison to the Veh.-treated ones. To test whether this increase in mitochondrial mass was accompanied by an increase in mitochondria number, mtDNA levels were determined by qRT-PCR. Despite the increased mitochondrial mass induced by MIM-10, no difference in mtDNA copy number was observed (Figure 3C,D).

Overall, this body of data suggests that the treatment may increase the mass of mitochondria without affecting the number of the cellular mitochondrial population in these cells under the tested conditions.

### 3.3. MIM-10 Affects the Activity of Enzymes Related to Mitochondrial Function Within HT-29 and SW620 Cells

**Figure 4:**
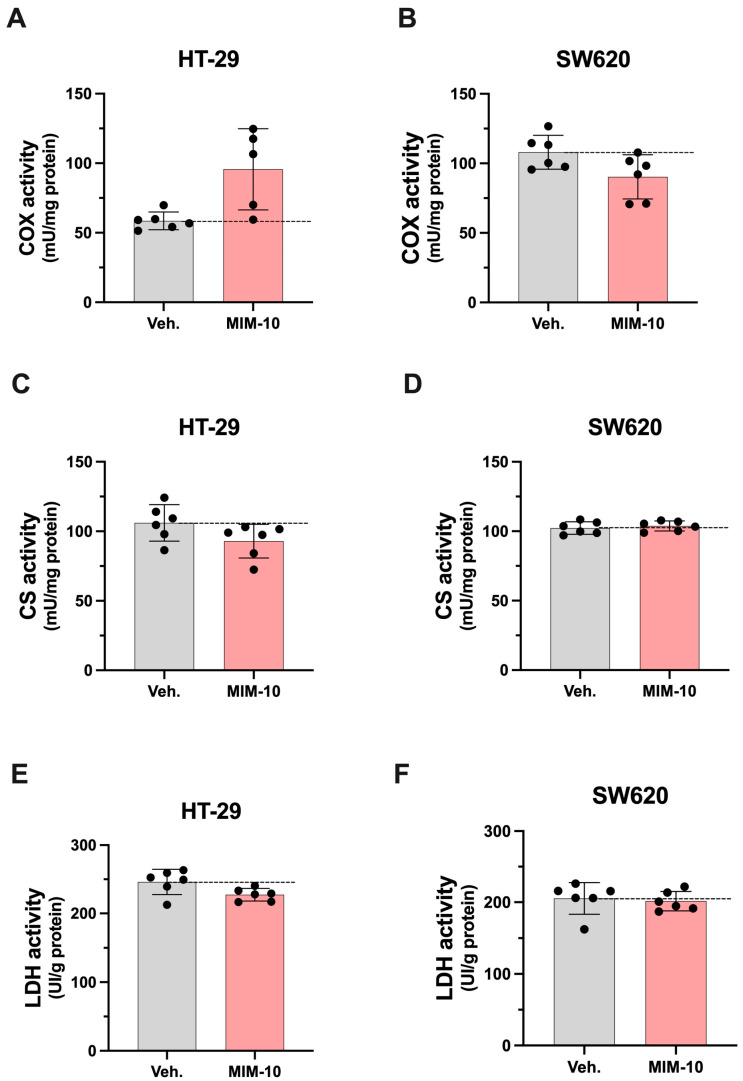
Effect of 20-day MIM-10 treatment on COX (**A**,**B**), CS (**C**,**D**), and LDH activity (**E**,**F**) in the HT-29 and SW620 colon cancer cell lines, respectively. Data are expressed as mU/mg of protein and presented as means ± S.D. from *n* = 6 replicates. The dotted lines are drawn to highlight the effects of MIM-10 compared with the Veh. treatment.

The effect of MIM-10 on enzyme activities related to energy metabolism and mitochondrial function was determined by measuring the activity of COX, CS, and LDH within the HT-29 and SW620 CRC cell lines (Figure 4).

As presented in Figure 4A,C,E, our experimental setting allowed us to appreciate that the MIM-10-treated HT-29 cells displayed higher COX activity and lower CS and LDH activities. The results related to the effects within SW620 differ from the ones observed in HT29. Indeed, lower COX activity was observed in SW620 compared to the Veh.-treated cells, and no effect was reported in CS and LDH activities (Figure 4B,D,F). In addition, the protein levels of the OXPHOS complex were determined by Western blotting. An analysis of the expression of CI, CII, CIII, CIV, and CV was thus conducted. According to our measures, MIM-10 induced changes in the expression levels of the five OXPHOS complexes, and mostly down-regulatory effects were reported in both cell lines (Supplementary Figure S3). Further research is needed before drawing any conclusions on how and to what extent MIM-10 affects mitochondrial function at molecular levels. Overall, these results, while very preliminary, suggest that MIM-10, by affecting COX, CS, and LDH activity, could potentially influence the mitochondrial function and energy metabolism of CRC cells.

Taken together, the results of this study suggest that MIM-10 could slightly decrease the cellular viability, reduce ROS production, and affect mitochondrial-related enzyme activity, potentially altering the metabolic and energetic profiles of non-metastatic and metastatic CRC cells.

## 4. Discussion

Current medical science shows that 90% of chronic diseases are associated with altered mitochondria, including neurodegenerative, age-related, and metabolic diseases, various forms of cancer, and other pathologies [37,38].

Cancer cells offer a valuable model for probing mitochondrial function owing to their unique metabolic shift, termed the Warburg effect, wherein they predominantly rely on glycolysis for energy generation, even in the presence of oxygen, as opposed to the oxidative phosphorylation typical of normal cells [39,40]. This metabolic alteration places substantial demands on cancer cell mitochondria, making them a pertinent system for exploring their involvement in tumorigenesis and therapy resistance. Investigating mitochondria in cancer cells can yield crucial insights into potential therapeutic avenues for disrupting their metabolism and curtailing tumor growth.

Regarding the rationale of the study and the choice of the two CRC models, the non-metastatic HT-29 cells and the metastatic SW620 cells, it is important to remember that these cells are highly proliferative cells in which mitochondrial metabolism plays a huge role [30,41]. Thus, with the increased recognition of the significance and diversity of cancer metabolism, metabolic reprogramming has become recognized as a hallmark of cancer [42], as these cells can dynamically integrate cellular signaling pathways and finely regulate their fitness in response to the environment and anti-cancer drugs [43]. In addition, these cells are also suitable for long-time culture [44]. In the context of this study, long-term cell culture is interesting, as it provides a consistent and stable platform for studying cellular processes over extended periods, allowing us to investigate “chronic” effects and the stability of these effects. Additionally, this cell ability facilitated large-scale experiments and MIM screening due to their ability to continuously proliferate in vitro, an advantage that has been leveraged here to complement our first study about MIM-10 [27]. Taking all these considerations into account, we were interested in evaluating the effects of MIM-10 after a long-course cell treatment. Thus, the reactive oxygen species (ROS)-dependent CRC cell lines were treated for 20 days with either the vehicle (Veh.) or MIM-10, by cell splitting and medium renewing every other day.

In the first part of the results, we observed that MIM-10 treatment impacted the cell viability of both cell lines after 20 days of treatment. Viability and ROS production are two parameters that are closely linked. The ROS generation constitutes a finely tuned process in tumor cells, as these species are highly implicated in cancer cells’ biology, from cell survival and progression to immune system escape and metastatic spreading [45]. Indeed, the generation of ROS, such as O_2_^−^ and H_2_O_2_, is a part of normal mitochondrial function [46] and is of paramount importance in both physiological functions and tumor development [45,47,48]. For this concern, it has been demonstrated that ROS, specifically H_2_O_2_, significantly increased the proliferation and migration of CRC-derived cell lines, including SW620 cells, when cells were exposed to concentrations ranging from 0,1 to 25 μM [14]. Thus, assessing the effect of MIM-10 on the proliferative, migration, and invasion capacities has to be considered in further research. Interestingly, MIM-10 treatment induced a decrease in the ROS production by about 10% in both CRC cells compared to Veh.-treated cells. This effect on viability, while relatively modest for both cell lines, suggests that the MIM-10 mechanism of action may not be directly inducing cytotoxicity. Instead, MIM-10 may exert its effects through modulating oxidative stress and mitochondrial function, potentially disrupting cancer cell metabolism and growth over time without causing cell death. These data are in line with previously published results, as MIM-10 was also able to slightly reduce the mitochondrial ROS production in immune cells derived from human PBMCs [27]. Overall, these results related to the effect of MIM-10 in reducing ROS production in both types of cells may be explained, at least partially, by the presence of tumor growth factor-β (TGF-β) (15 CH) in MIM-10, as this factor has been reported to be involved in ROS production. Studying the effect of the solely active substance TGF-β (15 CH) on ROS production would provide experimental-based knowledge and confirm, or not, our hypothesis. Concerning the role of TGF-β, it is important to report here that a particular crosstalk has been described between this cytokine and the levels of ROS. It has been shown that TGF-β modulates ROS production, thereby inducing redox imbalance in cancer, while ROS can, in turn, activate TGF-β [45,47]. The role of TGF-β in cancer progression is extensively studied; depending on the cell context and stage of cancer, it exerts different and contrasting effects. Indeed, TGF-β acts as an anti-tumorigenic signal at early stages, while at later stages, it exerts a pro-tumorigenic function by promoting cancer cell dissemination and metastasis [49,50].

The action on interleukin-2 (IL-2) could also have played a role in the observed effects of MIM-10. Indeed, previously published preclinical data reported that MIM-10 reduced the intra- and extra-cellular expression of IL-2 in PBMCs [27]. Looking at MIM-10 composition, it is interesting to notice that MIM-10 employs (i) SNA^®^-MIREG (16 CH), a SNA^®^ designed to target interleukin IL-2, and (ii) IL-2 (10 CH). Knowing that IL-2 promotes the growth and survival of specific tumor cells [51], it is possible that the employment of these two actives at ULDs in MIM-10’s formulation might have modulated IL-2 expression and played a role in the observed reduction in the viability of the two treated cell lines. However, only experimental-based evidence using unitary MI products employing the two above-mentioned active substances can confirm, or not, our hypothesis.

According to our results, MIM-10 also appears to affect mitochondria-related features, with these organelles being crucial for cellular function and response to signals. Considering that mitochondrial function and behavior are dynamic in response to cellular signals [52], mitochondria continuously undergo remodeling and experience fusion and fission processes, biogenesis, and degradation based on cellular needs. This flexible nature has been linked to the idea that mitochondrial respiration and metabolism may be spatially and temporally regulated by the organelle’s architecture and positioning [52,53]. Consequently, mitochondria are complex cellular organelles that can vary in number and machinery, and given that mitochondrial DNA (mtDNA) levels are dependent on the number of mitochondria, addressing mitochondrial dynamics can provide more data on how the tested medicine could influence cancer cell viability through mitochondria regulation [54]. Thus, with regard to these parameters, MIM-10 seemed to augment the mitochondrial mass index, a parameter indicating an increased mitochondrial biogenesis process without concurrent degradation, while it did not affect mtDNA levels. These results, together with the ones discussed above, could indicate that MIM-10 might be able to reduce the cell viability of HT-29 and SW620 cells by reducing the production of ROS in a manner that avoids mitochondrial degradation. On the contrary, the maintenance of mitochondrial DNA stability, which correlates with antioxidant functions, under MIM-10 treatment has a positive connotation when transferred to a CRC patient, as it would imply that the treatment could have the potential to affect cancer cell viability while preserving normal cellular functions [55,56]. Additional studies in non-cancerous colon cancer cells would provide insightful data about the MIM-10’s effect on cellular viability, addressing crucial safety and CRC-specific aspects. Moreover, such studies on mitochondrial function in non-cancerous cells from colon epithelium could provide insights into the fundamental metabolic processes and bioenergetic pathways essential for maintaining epithelial homeostasis, potentially revealing targets for preventing colon-related diseases. Understanding these mechanisms could also enhance our knowledge of how mitochondrial dysfunction contributes to a range of disorders beyond cancer and to what extent the 2LMIREG MI medicine could bring value in their prevention/treatment.

In addition, considering that mtDNA alterations have been associated with CRC progression [57,58,59] and therapy resistance [60], our preliminary results suggest that MIM-10 does not impact mtDNA. If confirmed by further experiments, our investigations about the effect of MIM-10 on mtDNA could allow for heightened tumor specificity, ensuring that the unique molecular characteristics of CRC are targeted, potentially enhancing treatment efficacy while minimizing side effects. By preserving normal cellular functions, the risk of untargeted effects to the tumor surrounding healthy tissues could be reduced, thus maintaining overall cellular health. Moreover, as cancer treatments often cause oxidative stress and systemic side effects such as fatigue and muscle weakness [61], adjuvant therapies aiming at limiting these effects would be very helpful.

The effect of MIM-10 treatment on the activity levels of enzymes related to energy metabolism and mitochondrial function, such as COX, CS, and LDH, were also assessed within the same CRC models. In a cancer cell, the measurement of these enzymatic activities provides insights into cancer metabolism and cellular functions. Lactate dehydrogenase plays a role in anaerobic glycolysis, and increased LDH activity is observed in cancer cells due to their preference for glycolytic metabolism [30]. The decrease in LDH activity observed in MIM-10-treated HT-29 cells could be seen as a valuable effect in the context of CRC and, if confirmed by further in vitro and in vivo studies, will open interesting therapeutic avenues for this MIM. Citrate synthase is involved in the citric acid cycle, and measuring its activity reveals information about mitochondrial function and oxidative metabolism in cancer cells [62]. Cytochrome-c oxidase, an enzyme in the mitochondrial electron transport chain, is crucial for oxidative phosphorylation [63]. By measuring these enzyme activities, valuable information about cancer cell metabolism, energy production, and mitochondrial function can be obtained. Interestingly, in the HT-29 cell line, the reduction in LDH activity was also accompanied by lower CS activity. By reducing their activity, MIM-10 might interfere with the Warburg effect of CRC, characterized by the marked increase in glucose uptake and lactate production, affecting their rapid cell growth. Conversely, the increase in COX activity reported in MIM-10-treated HT-29 cells could be explained as a compensatory response aimed at maintaining energy efficiency and sustaining the characteristic high energy demand of these CRC cells.

In the SW620 cell line, the results show a slight inhibition of the COX activity, while no changes were reported in LDH and CS activity. Reduced COX activity could mean that the mitochondrial electron transport chain might be less efficient [64], thus affecting the cells’ ability to proliferate.

Further studies are ultimately needed to explore the effects of MIM-10 on the proliferative capacity of CRC cells, not only after long-term culture treatment but also under short-term conditions.

If confirmed by further investigations, the decrease in COX activity observed in MIM-10-treated SW620 cells could be strategically valuable for several compelling reasons. Indeed, as COX is integral to cellular respiration, enabling ATP synthesis in the final step of the electron transport chain, by reducing its activity, ATP production could be impaired, effectively starving cancer cells of the necessary energy for rapid growth and proliferation. For this concern, assessing other parameters that evaluate the effect on mitochondrial respiration, such as the oxygen consumption rate, would be crucial. In addition, starvation and chemotherapy have been shown to induce the disconnection of the mitochondrial respiratory chain and an increase in oxidative stress in CT26 colon carcinoma cells [65].

Our COX activity results and our ROS could be linked together, as, after a 20-day treatment period in SW620 colon cancer cells, a decrease in COX activity could ultimately have led to a reduction in ROS production due to several adaptive mechanisms. COX, as a complex IV in the mitochondrial electron transport chain, is responsible for the final transfer of electrons to oxygen. When COX activity is initially inhibited, there may be an accumulation of electrons at upstream components such as CI and CIII, leading to increased ROS production as these electrons leak and react with oxygen.

However, over a prolonged treatment period like 20 days, SW620 colon cancer cells can undergo adaptive responses. For this concern, kinetic studies would be necessary to study the adaptations of CRC cells to MIM-10 treatment over time.

In summary, the overall results of the study indicated that MIM-10 treatment led to a decrease in cell viability and ROS production and an increase in mitochondrial mass, accompanied by changes in the activity of enzymes related to mitochondrial function such as COX, LDH, and CS. These results confirm the anti-oxidative effects of MIM-10 and open novel perspectives about the potential of this MI formulation on mitochondrial dynamics and oxidative stress management. However, it is important to highlight that this study presents several limitations that may affect the reliability and generalizability of the findings. As the experiment was solely performed once, one of the shortcomings is the absence of inferential statistical analysis, which is crucial for validating the results and drawing meaningful conclusions. To enhance the study’s power, future research should incorporate robust statistical methods to analyze the data, thereby providing a clearer interpretation of the results. Furthermore, from a model standpoint, the research focused solely on only two CRC cell lines, restricting the applicability of the findings to other cancer types or cell lines. Broadening the scope to include additional CRC cell lines and other cancer types could provide a more comprehensive understanding of the efficacy of the tested MIM. These findings, while preliminary, still have implications for understanding CRC cell metabolism regulation. Further studies are ultimately needed to confirm these effects on other CRC cell lines, as well as in vivo, before considering this MI formulation for clinical applications in the framework of CRC.

Moreover, considering that several of the active substances present in 2LMIREG, such as rh-IL-1β (10 CH), rh-IL-2 (10 CH), rh-TNF-α (10 CH), SNA^®^-HLA I (10 CH), SNA^®^-HLA II (10 CH), and finally, an rh-IL-2-targeting SNA^®^ at 16 CH, are also present in another MIM, 2LARTH, which displayed anti-inflammatory effects both in vivo and in vitro [17,18,66], it would be of great interest to further study MIM-10 to assess its effect in counteracting the secretion of pro-inflammatory mediators induced by cancer cells. An anti-inflammatory effect could be beneficial in some types of cancers as it can delay tumor progression [67,68].

## 5. Conclusions

The present manuscript reports for the first time the effects of one capsule of the MIM 2LMIREG, called MIM-10 throughout the manuscript, on several markers of mitochondrial metabolism in two in vitro models of CRC cell lines: the less invasive and non-metastatic HT-29 and the metastatic SW620 cell line. After being treated with MIM-10, these cells both showed a slight reduction in their viability and a diminution in their ROS production. This effect was accompanied by an increase in mitochondrial mass and changes in the activity of COX, LDH, and CS, three enzymes related to mitochondrial function. The overall results, while preliminary, suggest an antioxidant effect of MIM-10, which appeared interesting in the context of CRC, especially for those cancer cells such as SW620 cells that highly rely on ROS. If confirmed by further in vitro and in vivo studies, this body of results can provide novel pieces of evidence in the expanding field of MIM and in the understanding of CRC cell metabolism regulation.

## Data Availability

The data of the current study are available from the corresponding author upon reasonable request.

## Supplementary Materials

The following supporting information can be downloaded at https://www.mdpi.com/article/10.3390/life15050743/s1. Supplementary Figure S1: Effect of a 20-day 2LMIREG sequential treatment on cell viability (A,C) and mitochondrial ROS production (B,D) in non-metastatic HT-29 CRC cell line and metastatic SW620 CRC cell line. Data are expressed as a percentage relative to the Veh. group, which was set at 100%. Data are presented as means ± standard deviation (S.D.) from *n* = 12 replicates (A,D) and *n* = 6 replicates (B,D). The dotted lines are drawn to highlight the effects of MIM-10 compared with the Veh. treatment. Supplementary Figure S2: Effect of MIM-10 treatment at different time points (day 4, day 12, day 16, and day 20) in HT-29 and SW620 cell lines. Data are expressed as a percentage of viability relative to the untreated control (Ct), which was set at 100%. Data presented as means ± S.D. from *n* = 6 replicates. Supplementary Figure S3: Effect of 20-day MIM-10 treatment on protein levels of OXPHOS complexes of HT-29 and SW620 colon cancer cell lines. Data are expressed as a percentage of expression relative to the untreated control (Ct), which was set at 100%. Data are presented as means ± S.D. from *n* = 6 replicates.

## Abbreviations

The following abbreviations are used in this manuscript: ATCC: American type culture collection; BCA: bicinchoninic acid; CI: complex I; CII: complex II; CIII: complex III; CIV: complex V; CV: complex V; CH: centesimal Hahnemannian; COX: cytochrome-c oxidase; CRC: colorectal cancer; CS: citrate synthase; DMEM: Dulbecco’s modified Eagle’s medium; DMSO: dimethyl sulfoxide; DNA: deoxyribonucleic acid; EDTA: ethylenediaminetetraacetic acid; EMT: Epithelial-Mesenchymal Transition; FBS: fetal bovine serum; GAPDH: glyceraldehyde-3-phosphate dehydrogenase; HLA: human leukocyte antigen; HLA-DP: Human Leukocyte Antigen—DP isotype; HLA-I: Human Leukocyte Antigen Class I; HLA-II: Human Leukocyte Antigen Class II; IL: interleukin; LD: low doses; LDH: lactate dehydrogenase; MI: micro-immunotherapy; MIM: MI medicine; MTG: MitoTracker^VR^ Green; mtDNA: mitochondrial DNA; OXPHOS: oxidative phosphorylation; PGE2: prostaglandin E2; RT-PCR: real-time reverse transcription-polymerase chain reaction; RA: receptor antagonist; rh: recombinant human; RNA: ribonucleic acid; ROS: reactive oxygen species; S.D.: standard deviation; SNA^®^: specific nucleic acids; TBS-T: tris-buffered saline; TGF-β: transforming growth factor-beta; TNF-α: tumor necrosis factor alpha; ULD: ultra-low doses; UK: United Kingdom; USA: United States of America; Veh.: vehicle, WB: Western blot.
