# Supplementary figures and images for: Active Substances from the Micro-Immunotherapy Medicine 2LMIREG Display Antioxidative Properties In Vitro in Two Colorectal Cancer Cell Lines
Source: Life (Basel). 2025 May 6;15(5):743. doi: 10.3390/life15050743 (PMC12112867; doi:10.3390/life15050743)

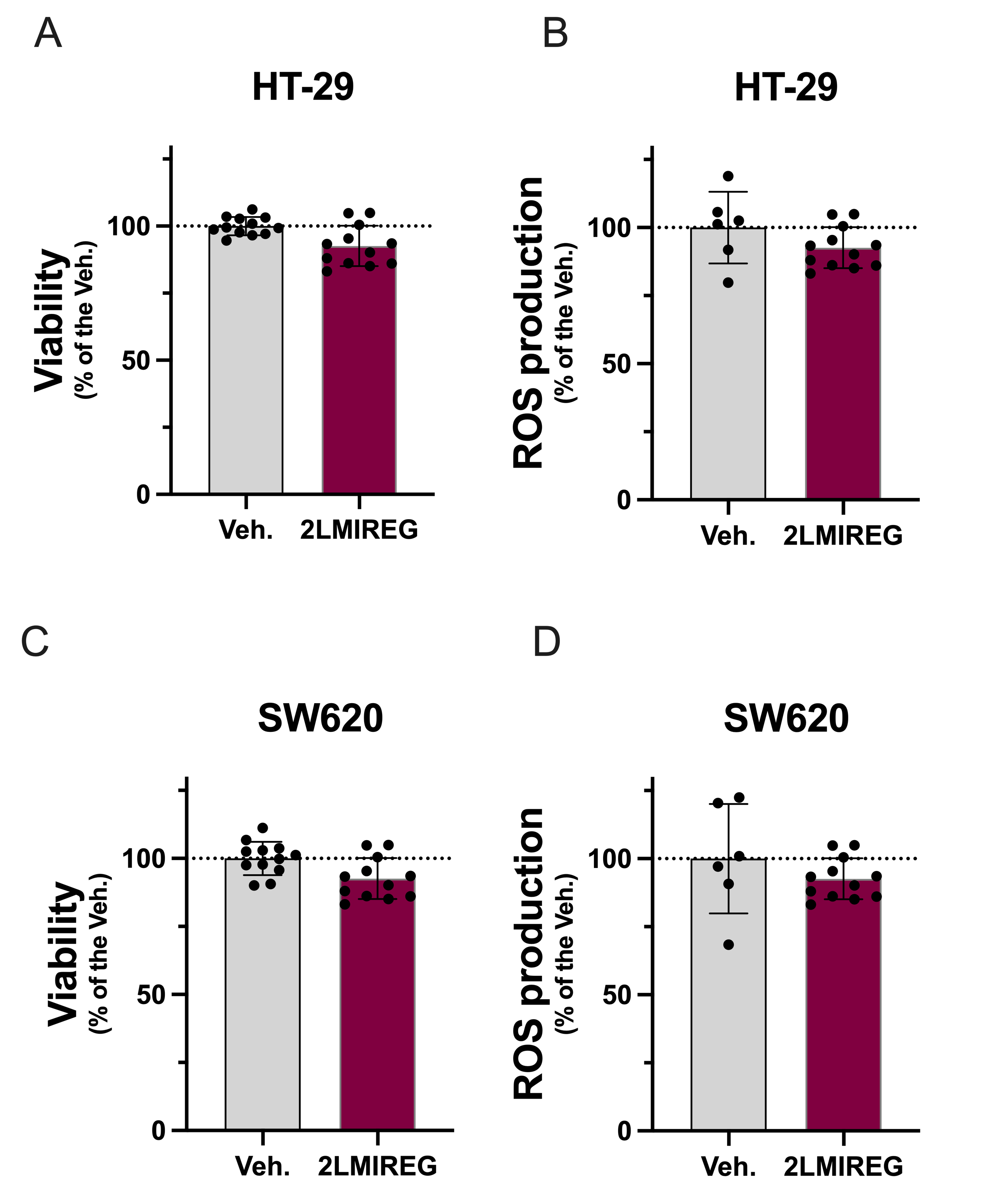

Supplement: Supplementary file 1 [file life-15-00743-s001.zip › Supplementary Figure S1.tiff]

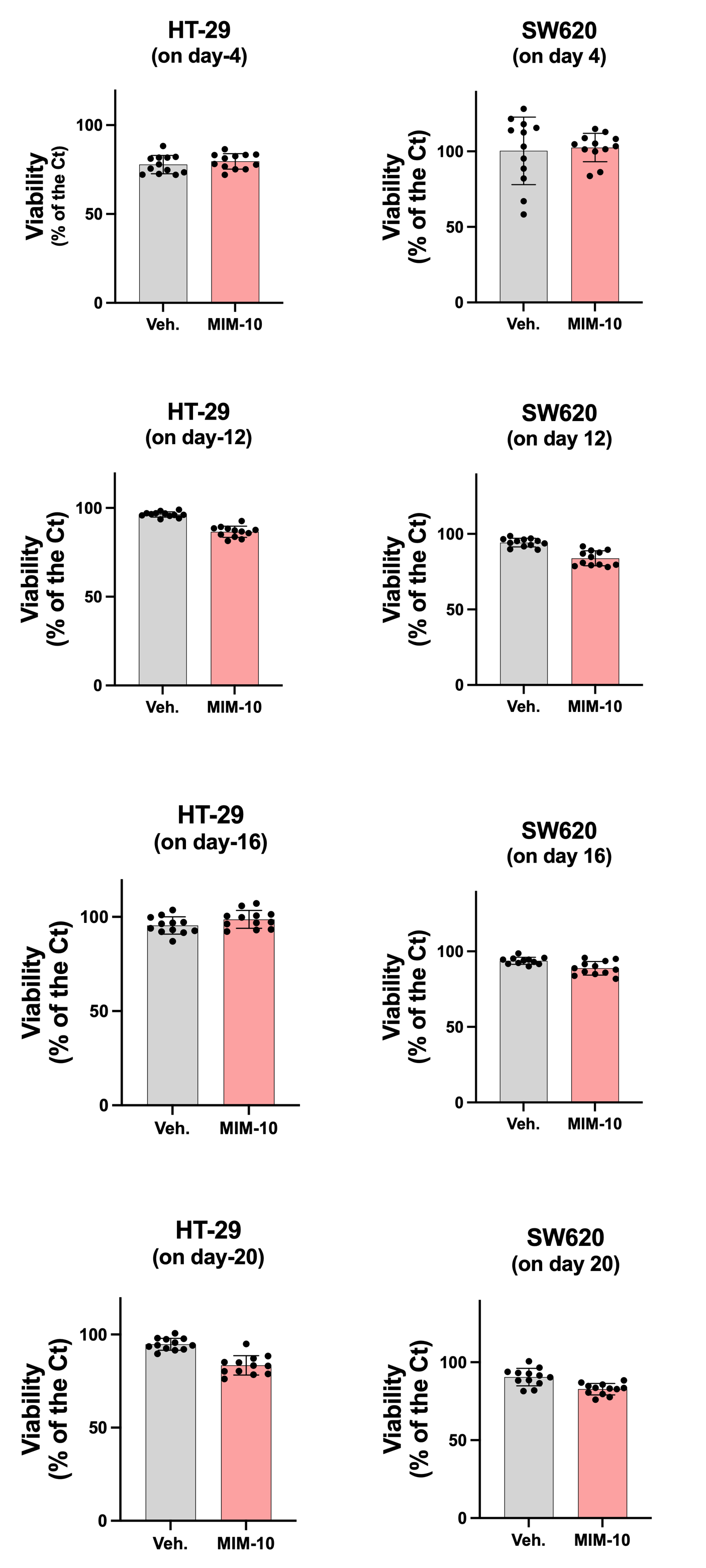

Supplement: Supplementary file 1 [file life-15-00743-s001.zip › Supplementary Figure S2.tiff]

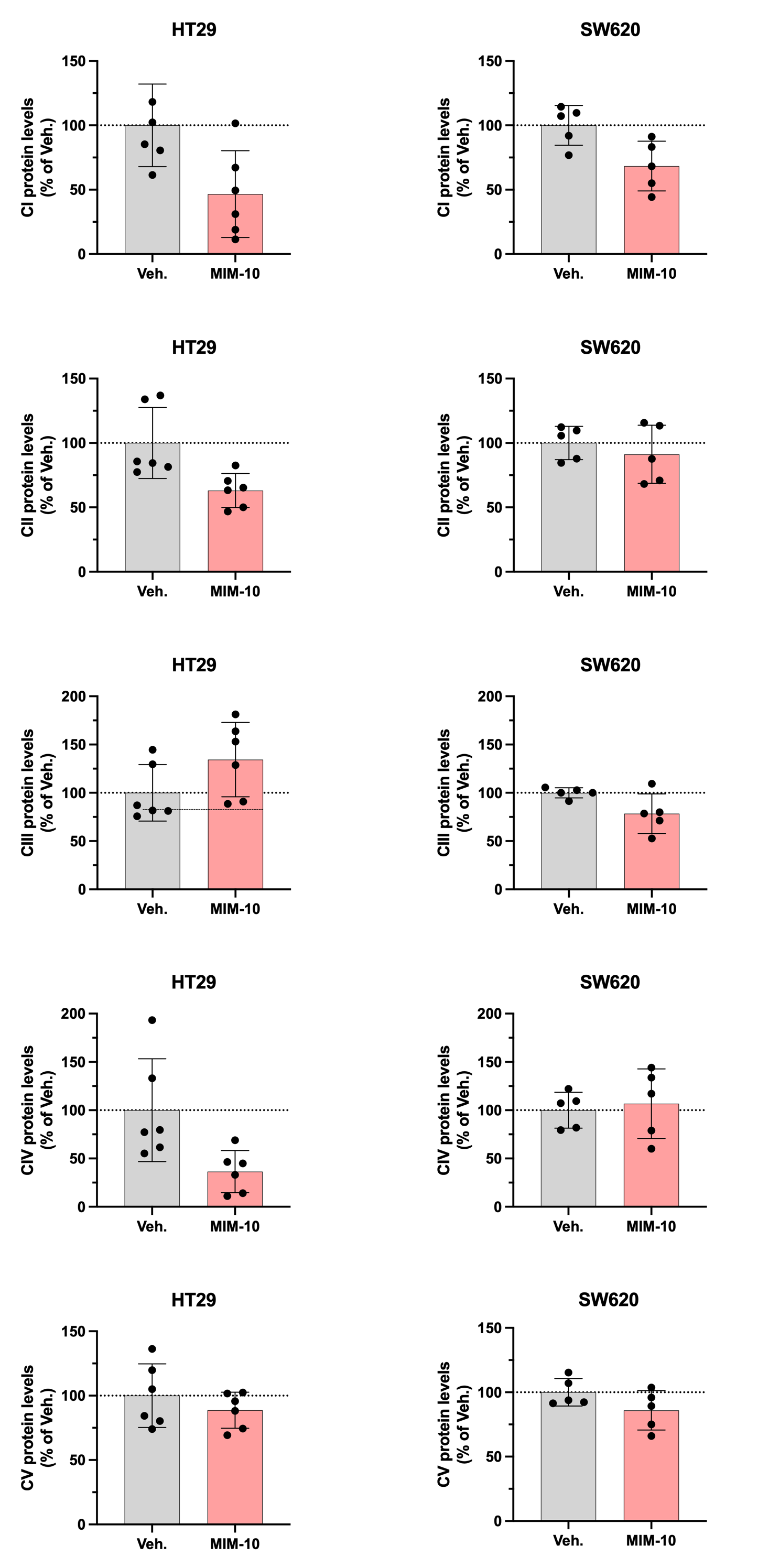

Supplement: Supplementary file 1 [file life-15-00743-s001.zip › Supplementary Figure S3 new version.tiff]
